# Reproductive Health in Rheumatology: Making ‘Early and Regular’ Work in Practice

**DOI:** 10.1016/j.ero.2025.06.001

**Published:** 2025-07-07

**Authors:** Luis Fernando Perez-Garcia

**Affiliations:** Department of Rheumatology, Erasmus MC, University Medical Center, Rotterdam, the Netherlands

## Abstract

Reproductive health is increasingly recognised as a core aspect of rheumatology care, particularly for patients of reproductive age living with chronic inflammatory conditions. Both the newly updated EULAR recommendations and the 2020 American College of Rheumatology guideline call for reproductive health to be addressed ‘early and regular’ or ‘early and often’ in clinical encounters. However, the practical implementation of this guidance remains a challenge. In daily practice, discussions about fertility, contraception, and family planning are often delayed, inconsistent, or omitted entirely, especially for male, trans, and nonbinary patients. This viewpoint outlines a practical approach to integrating reproductive health into routine rheumatology care using 2 complementary strategies: the Permission, Limited Information, Specific Suggestions, Intensive Therapy (PLISSIT) model and a communication approach referred to as ‘Inform and Acknowledge’. The PLISSIT model provides a scalable framework for initiating and tailoring reproductive health conversations, while 'Inform and Acknowledge' offers a rhythm for maintaining those discussions over time, even when they are not immediately relevant. These strategies are designed to be flexible, time-sensitive, and inclusive of diverse reproductive identities and needs. The article also highlights structural barriers, including limited consultation time and lack of training, and calls for more implementation research to support integration of reproductive care into real-world rheumatology practice. By shifting from reactive, risk-focused conversations to proactive, informed planning, clinicians can support patients in making timely and empowered reproductive health decisions. Making reproductive health routine is not just an ethical imperative—it is part of providing complete, patient-centered rheumatologic care.

New European Alliance of Associations for Rheumatology (EULAR) recommendations on reproductive health in rheumatic and musculoskeletal diseases were recently published, emphasising the importance of addressing reproductive health ‘early and regular’ in clinical care [1]. This aligns closely with guidance from the American College of Rheumatology, which calls for reproductive health to be discussed ‘early and often’ [2]. While the language differs slightly, the message is the same: these conversations need to start sooner and happen more consistently.

These are essential and long-overdue shifts. However, in daily clinical practice, the challenge is not just what to discuss,it’s also how, when, and how often. Without practical tools, even the best recommendations risk staying on paper.

We need practical strategies that make reproductive care a routine part of rheumatology—for all our patients. I use the following 2: the Permission, Limited Information, Specific Suggestions, Intensive Therapy (PLISSIT) model and an approach I call '*Inform and Acknowledge'*. They are not complicated. They work. And they make this part of care real.

## THE PROBLEM WITH ‘EARLY AND REGULAR’

I work in reproductive rheumatology, and I hear this all the time: ‘You should be talking about reproductive health early and regular’. But no one tells you what that looks like in clinic. Not the guidelines. Not the systems. And definitely not our training [3,4].

So here is what usually happens: one conversation at diagnosis—maybe. Alternatively, a quick mention when starting methotrexate. After that? Silence, unless the patient brings it up [5,6].

We prescribe medications that affect fertility, pregnancy, and parenting. We see patients during their key reproductive years. However, we do not routinely talk about contraception, fertility, or future planning. And when we do, we often default to the assumption that reproductive health only applies to women—and only when they want to get pregnant.

This is not just a communication issue. It is a care gap. Moreover, it is not an imaginary gap, there is evidence. Studies show that many women with inflammatory arthritis or systemic lupus erythematosus report that reproductive issues were never discussed or came up too late [7,8]. Others were not warned about medication-related fertility risks or pregnancy safety until they asked directly—if they asked at all [9].

It is not just women who are affected. In our own work, we have found that men are frequently overlooked in reproductive conversations [10,11]. Cyclophosphamide and sulfasalazine can impair fertility, yet male patients are often not informed [12]. Methotrexate, while not currently considered to affect fertility, has historically been a source of uncertainty [13]. Until recently, limited data led to mixed messages and inconsistent advice—leaving many men unsure about their reproductive options. And for trans and nonbinary patients, the silence is even louder.

‘Early and regular’ is supposed to fix this. However, without a framework, it becomes just another vague phrase.

## A WAY FORWARD: PLISSIT

One model that helps is **PLISSIT**, originally developed for sexual health but highly adaptable to reproductive care in rheumatology. It gives clinicians a structured way to introduce sensitive topics without needing to be an expert or spend much time. The acronym stands for:

• **Permission:** Invite the patient to talk about the topic, and signal that it is welcome.
• **Limited Information:** Offer 1 or 2 relevant facts, tailored to the situation.
• **Specific Suggestions**: Provide actionable guidance or next steps.
• **Intensive Therapy**: Refer to specialists if more in-depth support is needed.

You do not need to go through all 4 steps every time. Even just giving **Permission**—letting patients know it is okay to talk about fertility, contraception, or pregnancy—can open a door. The strength of PLISSIT is its flexibility: it works during busy visits, fits different patient needs. And crucially: it’s gender inclusive. It applies to men, women, nonbinary people—anyone with a reproductive system and a long-term condition.

## HOW I IMPLEMENT PLISSIT IN DAILY PRACTICE: INFORM AND ACKNOWLEDGE

To apply PLISSIT over time, I use an approach I call '*Inform and Acknowledge'*: Inform early. Acknowledge regularly.

**Inform** early—usually at diagnosis or when starting a medication with reproductive implications.

*This medication may have side effects related to fertility, or it is not compatible with pregnancy. So even if it is not relevant right now, it is something we should keep in mind.*

**Acknowledge** again during regular care—especially during life changes, medication shifts, or disease activity changes.

But here is the key: *Acknowledge* does not just mean asking if family plans have changed. It means clearly stating how their disease or medication could impact their reproductive health.

*This medication can affect sperm quality. If you’re thinking about having children, let’s talk about it early.*

*Active disease itself can reduce fertility - something we consider when discussing family planning.*

Patients do not always see the connection. They might not link joint inflammation to fertility. They might not realise their medication could delay conception or that fertility preservation is even an option. By acknowledging the link, we help them make informed, timely decisions—before they face delays or regret.

‘Inform and Acknowledge’ is flexible and low effort. One sentence may be all it takes. The point is consistency, not perfection. Using this approach makes reproductive health a visible, ongoing part of care. It removes the burden from the patient to bring it up. It protects them from surprises. And it builds trust.

We must also be realistic: clinicians are stretched. Time in the consultation room is limited. We are juggling active disease, labs, comorbidities, treatment changes—all of it. The expectation to cover reproductive health—on top of everything else—can be overwhelming. That is why we need simple tools, more training, but also structural support. Electronic prompts, nurse-led check-ins, patient education materials—these are all options worth exploring to embed reproductive health more efficiently in our real-world care. To support this, Table 1 provides a set of practical recommendations with associated clinical impacts to guide implementation.

**Table 1:** Recommendations for enhanced clinical impact

**Recommendation**	**Expected clinical impact**
Embed PLISSIT prompts into EHRs	Standardises reproductive health discussions; ensures they are addressed systematically across consultations
Include reproductive health screening in nurse-led or intake assessments	Expands discussion beyond physician encounters; increases screening coverage
Add prompts to treatment protocols (eg, methotrexate, mycophenolate, cyclophosphamide)	Aligns reproductive counselling with high-risk medication initiation points
Implement reproductive health alerts based on patient age or life stage	Encourages timely discussions beyond treatment triggers (eg, age <40, postpartum, partner change)
Use structured EHR templates to document reproductive counselling	Supports efficiency and consistency; ensures key topics are addressed and recorded
Ensure that clinicians and other health care professionals receive training in reproductive health communication across gender identities and life stages	Builds confidence and skills to address reproductive health with patients of different genders and at varying stages of life
Develop brief patient education tools (eg, leaflets or digital prompts)	Facilitates patient-initiated discussions; normalises reproductive health as part of chronic disease care

EHR, electronic health record; PLISSIT, Permission, Limited Information, Specific Suggestions, Intensive Therapy.

Research on which options actually work in different settings and how to integrate this into busy clinics is urgently needed. Implementation science should be part of how we close this gap—not just more recommendations.

## CHANGING THE CULTURE: SHIFTING FROM REACTIVE TO PROACTIVE

It is also time to change the culture. The old pattern sounds like this: ‘Miss X is pregnant on methotrexate—what now?’ That is reactive and high-risk. Instead, let’s flip the script: ‘Miss X is 25, starting methotrexate, and has no children yet. I will take a minute to bring up fertility’. The PLISSIT model provides a simple structure to help clinicians move from reactive to proactive care. Table 2 illustrates how this can be applied in practice.

**TABLE 2:** PLISSIT model applied to reproductive health in rheumatology

**Step**	**Explanation**	**Example in clinical practice**
Permission	Invite the patient to talk about reproductive health; signal that the topic is welcome and appropriate.	‘I would like to ask you a few questions about fertility and pregnancy, if that is okay with you’.
Limited information	Share brief, relevant facts tailored to the patient’s context.	‘This medication is not compatible with pregnancy, and it may affect fertility. It is important to plan ahead’.
Specific suggestions	Offer actionable guidance, planning steps, or referrals.	‘If you are thinking about having children in the future, we can adjust treatment now to keep that option open’.
Intensive therapy	Refer to specialists (eg, reproductive medicine, fertility preservation) for more complex cases.	‘Before starting cyclophosphamide, I recommend a fertility consultation to discuss sperm or egg preservation’.

PLISSIT, Permission, Limited Information, Specific Suggestions, Intensive Therapy.

This is not just about managing risk—it is about supporting informed, timely decisions around reproductive health and family planning. That is the shift we need.

## CONCLUSION

‘Early and regular’ cannot just be a line in the guidelines. It has to shape how we prescribe, plan, and communicate about reproductive health. PLISSIT and Inform and Acknowledge offer a way to make that real—today, in real clinics, with real patients. It is time to stop waiting and start doing.

## CRediT authorship contribution statement

**Luis Fernando Perez-Garcia:** Writing – review & editing, Writing – original draft, Conceptualization.

## Competing interests

LFPG reports a relationship with Erasmus University Medical Center that includes employment.
